# Overcoming resistance in HER2-positive gastric cancer: a case report on the synergistic effect of pembrolizumab and trastuzumab

**DOI:** 10.1097/MS9.0000000000002614

**Published:** 2024-10-11

**Authors:** Ting Gong, Qing Ma, Yaoyao Ren, Liyan Gu, Hui Lv, Diansheng Zhong

**Affiliations:** Department of Medical Oncology, Tianjin Medical University General Hospital, Tianjin, China

**Keywords:** case report, drug resistance, immune checkpoint inhibitors, stomach neoplasms, trastuzumab

## Abstract

**Introduction:**

The first-line standard therapy for advanced HER2-positive gastric cancer is chemotherapy combined with trastuzumab and pembrolizumab, while pembrolizumab alone does not benefit as a monotherapy in patients with mismatch repair proficiency (pMMR). This case explores the therapeutic potential of adding pembrolizumab to patients who were resistant to trastuzumab, focusing on the synergistic effect of an immune checkmate inhibitor, combined with HER2 antibody.

**Case Presentation:**

An 87-year-old metastatic gastric cancer patient, whose medical history was significant for intolerance to chemotherapy and had a poor status of performance. Immunohistochemical staining was presented as HER2 (3+), pMMR, and PD-L1 was 4. Initially treated with trastuzumab monotherapy, the patient showed no response and experienced progressive disease. Subsequently, a combined regimen of trastuzumab and a half-dose of pembrolizumab was administered every 3 weeks. Remarkably, it led to a significant reduction in tumor size, achieving partial remission (PR) after two cycles. This response was sustained over 21 months, as evidenced by the latest CT scans.

**Clinical Discussion:**

The concurrent administration of trastuzumab and pembrolizumab has demonstrated synergistic antitumor activity, achieving clinical efficacy in cases where each agent alone proved ineffective. Preclinical studies illustrated that tumor regression induced by HER2 antibodies requires T cell involvement, and the combination of immune checkpoint inhibitors with trastuzumab augments HER2-specific T cell responses, promotes immune cell recruitment, and induces the expansion of peripheral memory T cells, which showed synergistic rationales for a combination of pembrolizumab and trastuzumab.

**Conclusion:**

The observed synergy between pembrolizumab and trastuzumab highlights a promising treatment avenue that warrants further investigation.

## Introduction

Highlights

- In this case, an elderly patient with HER2-positive, pMMR gastric cancer, who initially did not respond to trastuzumab, experienced significant tumor shrinkage and prolonged PFS after the addition of a half-standard dose of pembrolizumab. This response has been sustained for 21 months without progression to date.
- Unconventional application: Despite typically being adjuncts to chemotherapy, a combination of trastuzumab and pembrolizumab was applied in a rare clinical scenario where trastuzumab was not effective alone.
- Synergistic effect: The patient, already resistant to HER2-targeted therapy and classified to the pMMR population (for whom immunotherapy alone is ineffective), showed a significant response to a half-dose of pembrolizumab combined with trastuzumab, underscoring a notable synergistic effect.
- Clinical insight: This case provides valuable insights into alternative therapeutic strategies for patients who are ineligible for standard chemotherapy regimens, suggesting potential for broader application in similar clinical settings.

For years, the cornerstone of first-line treatment for advanced HER2-positive gastric cancer has predominantly been chemotherapy, supplemented with targeted therapy using trastuzumab, an anti-HER2 monoclonal antibody^1^. The advent of immune checkmate inhibitors (ICIs) has introduced a potential to enhance the efficacy of treatments for HER2-positive gastric cancer, extending progression-free survival (PFS)^2^. However, the benefits of ICIs as a monotherapy are restricted to patients with mismatch repair deficiency (dMMR) or high microsatellite instability (MSI-H), leaving a significant portion of the patient population without this option. This raises an important question: Can patients, particularly those of advanced age or with poor performance status who are unable to tolerate chemotherapy, derive benefit solely from HER2-targeted therapy or a combination of targeted therapy and ICIs? This article presents the case of a patient unable to undergo chemotherapy, who initially did not respond to trastuzumab monotherapy but achieved partial remission (PR) upon the addition of ICIs. This case contributes to the evolving discourse on personalized treatment strategies, suggesting a potential pathway for patients who are ineligible for standard chemotherapy regimens.

## Case presentation

**Figure 1:**
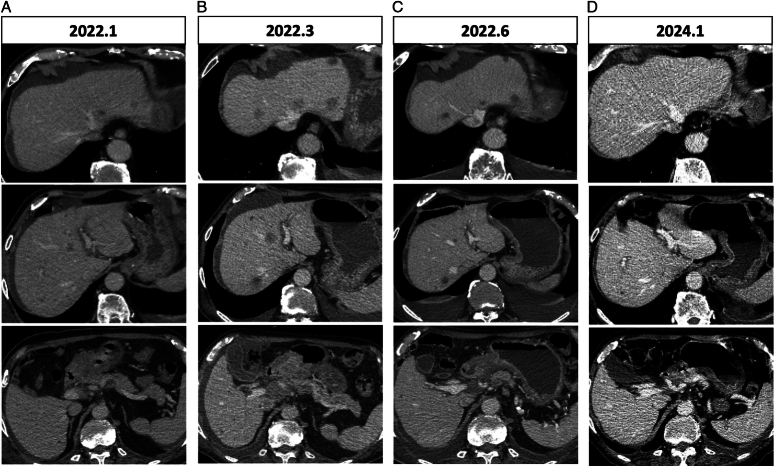
CT imaging during the clinical course. (A) Baseline image before the initiation of trastuzumab treatment. Hepatic nodules and antrum mass can be observed. (B) After one cycle of trastuzumab treatment, tumor progression was evidenced by increased tumor size. (C) After two cycles of the combination treatment of pembrolizumab and trastuzumab, the tumor began to shrink. (D) Latest CT image taken after 21 months of combined treatment with trastuzumab and pembrolizumab showed significant tumor reduction.

An 84-year-old man with melena in our hospital was referred for gastroscopy in 2019. Biopsy findings from the gastric angle confirmed gastric adenocarcinoma, with immunohistochemical staining revealing positivity for CK19, CEA, MSH2, MSH6, PMS2, MLH1, partial positivity for CK7, strong positivity for HER2 (3+), and high proliferative index (Ki-67 at 80%). He denied having a history of smoking or drinking, as well as any other medical or family history. Opting against surgery and suffering from an intolerance to the oxaliplatin and S-1 (SOX) combination after just one cycle, the patient experienced intermittent melena and hematemesis, which were temporarily alleviated with proton-pump inhibitors (PPIs), hemostatic measures, and fluid rehydration. In December 2021, he experienced more severe hematemesis and critical anemia, with a hemoglobin level of 42 g/l. Emergency surgery to address bleeding and pyloric obstruction included exploratory laparotomy, jejunostomy, and peritoneal lymph node biopsy, which confirmed metastasis in the greater curvature lymph nodes (station number 4) but was not involved in the greater omentum. Unfortunately, melena recurred one month after surgery (January 2022). A subsequent enhanced abdominal computed tomography (CT) showed slight enlargement of the gastric sinus mass and the perigastric and pancreatic lymph nodes, along with new hepatic nodules (Fig. 1A). Next-generation sequencing (NGS) revealed ERBB2 amplification (CNV=3.50), a high tumor mutation burden (TMB 20.70Muts/Mb), and low microsatellite instability (MSI-L) status. Additional genomic alterations of uncertain significance were identified in FLCN p.H429fs, MSH3 p.N385fs, TP53 p.W53*, and the Combined Positive Score (CPS) for PD-L1 was 4 (DAKO 22C3).

By this time, the patient, now 87, was in poor physical condition, ECOG=2, with severe anemia, hypokalemia, and hypoproteinemia (albumin 27 g/l). Due to pyloric obstruction, the patient was able to consume food only through a jejunostomy tube. The patient further declined chemotherapy and pembrolizumab treatment. Consequently, the patient was initially treated with monotherapy using trastuzumab (8  mg/kg loading dose, then 6  mg/kg) every 3 weeks intravenously, necessitated by his condition. One month later, a CT scan indicated progressive disease (PD), with increased size of the antrum mass, periantral lymph nodes, and intrahepatic nodules (Fig. 1B). Eventually, the patient consented to immunotherapy, but remained opposed to chemotherapy. We administered a combined regimen of trastuzumab and half a dose of pembrolizumab (100 mg) every 3 weeks. Surprisingly, with the addition of a checkpoint inhibitor, the tumor responded with a significant reduction in size. The patient achieved PR according to the Response Evaluation Criteria in Solid Tumors (RECIST) 1.1 after two cycles of treatment (Fig. 1C), and the response was sustained for 21 months, as confirmed by the latest CT assessment (January 2024, Fig. 1D). The patient had no complaints of discomfort and has regained the ability to consume food orally and has consequently discontinued the use of the jejunostomy tube for nutritional support. Since the expiration of the pembrolizumab charitable donation program in January 2024, the patient has declined CT evaluations for economic reasons and was receiving regular infusions of trastuzumab combined with a half-dose of pembrolizumab till 16 May 2024. From 7 June 2024, he switched pembrolizumab 100 mg to tislelizumab 200 mg every 3 weeks. As of the last follow-up on 24 July 2024, the patient was in good physical condition, with an ECOG performance status of 1, managing normal dietary intake without reliance on the jejunal stoma tube. By taking rabeprazole and iron supplements, the hemoglobin levels are maintained at 88 g/l. Transaminase levels, creatinine, and thyroid function tests are all within normal limits. The dosage of antihypertensive medication remains consistent, and blood pressure is well-controlled within the normal range.

## Discussion

ERBB2 (HER2 or HER2/neu), a tyrosine kinase receptor, is implicated in cell proliferation and is associated with reduced survival and worse prognosis when overexpressed in gastric cancer^3^. In the present case, the patient did not benefit from first-line monotherapy targeting HER2. This lack of efficacy could be attributed to the fact that trastuzumab is conventionally administered alongside chemotherapy in the treatment of gastric cancer. The ToGA trial^1^ demonstrated that the combination of trastuzumab with chemotherapy significantly improved outcomes in patients with HER2-positive/IHC 3+ gastric tumors, with a hazard ratio (HR) of 0.58, and an overall response rate (ORR) of 47%, compared to 35% with chemotherapy alone. Accordingly, the NCCN guidelines recommend trastuzumab in combination with a fluoropyrimidine and a platinum-containing drug as first-line treatment for HER2-positive (IHC-confirmed or FISH-confirmed) advanced gastric cancer.

In the context of gastric cancer, this elderly patient with tumors that were pMMR, MSI-L, and CPS of 4 did not typically fall into the group that benefits from ICIs. Based on single-arm studies^4–7^, the Food and Drug Administration (FDA) approved pembrolizumab for MSI-H/dMMR solid tumor patients who have failed front-line therapy and have no other treatment options, with an ORR of 39.6% for the overall population (*n*=149). The ORR was 56% in patients with cancer of the stomach or gastroesophageal junction. However, for the vast majority of microsatellite stable (MSS) or MSI-L, ICI attempts in the treatment of advanced gastric cancer have encountered many difficulties and failures and have stagnated in the process of first-line and second-line advancement.

Phase III clinical studies represented by the KEYNOTE-061^8^, KEYNOTE-062^9^, and ATTRACTION-4^10^ studies failed to show any advantage over conventional chemotherapy in either monotherapy or combination first-line or second-line ICIs. It was not until 2021, based on positive results from the CHECKMATE-649 study^11^, that nivolumab combined with chemotherapy was approved by the FDA for advanced gastric and gastro-esophageal junction adenocarcinoma (GC/GEJC) first-line treatment. Notably, all patients recruited to these clinical trials were HER2-positive excluded.

The standard treatment for advanced gastric cancer typically involves doublet chemotherapy as the foundation, supplemented by anti-PD-1 antibody, with or without anti-HER2-targeted drugs depending on HER2 expression. Generally, for patients intolerant to standard chemotherapy regimens, adaptations such as dose reductions, single-agent chemotherapy, or substitution with alternative chemotherapeutics like paclitaxel or irinotecan are considered. However, this particular patient steadfastly declined all forms of chemotherapy. Faced with the prohibitive costs of pembrolizumab, we initially administered monotherapy with a HER2-targeted agent as the first-line treatment.

Despite HER2 overexpression, the patient’s tumor did not respond to single-agent HER2-targeted therapy. The patient’s tumor was determined to be pMMR and MSI-L, indicating that the use of a PD-1 inhibitor alone might not be effective unless combined with chemotherapy to enhance treatment outcomes. Against all expectations, the addition of pembrolizumab effectively reversed resistance to trastuzumab. These unconventional therapeutic approaches not only achieved partial remission and substantially enhanced the patient’s quality of life but also yielded insights into the synergistic potential of targeted immunotherapy.

The patient’s disease demonstrated remarkable regression following the administration of a half-dose of pembrolizumab. We assume that the notable therapeutic response observed may be due to the synergistic effect of the PD-1 inhibitor and HER2-targeted agent. For patients with metastatic HER2-positive gastric cancer, the KEYNOTE-811 trial demonstrated that adding pembrolizumab to first-line therapy significantly improved PFS when used in conjunction with trastuzumab and chemotherapy^12^.

Previous studies have shown preclinical and clinical rationales for combination usage in HER2-positive disease. Trastuzumab facilitates the uptake of HER2 by dendritic cells, which is mediated by the Fc receptor and is specific to trastuzumab. This increased cross-presentation of E75, the immunodominant epitope derived from the extracellular domain of the HER2 protein, which is mediated by trastuzumab treatment, enables a more efficient expansion of E75-specific cytotoxic T cells (E75-CTL)^13^. Park *et al*.^14^ found in a mouse model that tumor regression by an anti-HER2/neu antibody is T cell-dependent. Chaganty *et al*.^15^treated syngeneic mouse tumors transduced to overexpress HER2 with the anti-human HER2 antibody trastuzumab, which upregulated PD-L1 expression. In monocultures, PD-L1 expression in HER2-overexpressing cancer cells was upregulated only when they were co-cultured with peripheral blood mononuclear cells. Stagg *et al*.^16^ demonstrated that anti-ErbB-2 mAb therapy of ErbB-2 tumors induced sustained tumor regression in syngeneic immunocompetent mice but failed to have an effect in mice depleted of CD8α+ cells. Moreover, the addition of anti-PD-1 mAb significantly improved the therapeutic activity of anti-ErbB-2 mAb.

Therefore, the synergy between targeted therapy and immunotherapy is likely crucial in this context. Notably, next-generation sequencing (NGS) results indicate that this patient may be more likely responsive to immunotherapy despite being categorized within the pMMR cohort, suggesting an atypical advantage from immunotherapeutic approaches in this subgroup. For example, this patient exhibits a high TMB and positive gene mutations associated with immunotherapy, including HLA heterozygosity, FLCN, and MLH3.

## Conclusion

This case illustrates the potential of combining PD-1 inhibitors with HER2-targeted therapies to overcome resistance in HER2-positive gastric cancer, suggesting a promising treatment strategy for patients who do not respond to traditional therapies. The observed synergy between pembrolizumab and trastuzumab warrants further investigation in clinical trials to define the optimal strategies for integrating ICIs into the treatment paradigm for HER2-positive gastric cancer. Our findings contribute to a growing body of evidence supporting the use of combined ICIs and targeted therapy in managing complex cases of gastric cancer, potentially guiding future therapeutic approaches.

## Ethical approval

All procedures performed in studies were approved by the Ethical Committee of Tianjin Medical University General Hospital.

## Consent

Written informed consent was obtained from the patient’s relative for publication of this case report and any accompanying images.

## Source of funding

No funding was received.

## Author contribution

T.G., Y.R., and Q.M.: conceptualization; T.G., Y.R., L.G., H.L., and Q.M.: data curation; T.G.: writing original draft; Q.M. and D.Z.: writing review and editing.

## Conflicts of interest disclosure

The authors declare no conflicts of interest.

## Research registration unique identifying number (UIN)

Retrospectively, case reports cannot be registered.

## Guarantor

Ting Gong and Diansheng Zhong.

## Data availability statement

The datasets generated and/or analyzed during the current study are publicly available.

## Provenance and peer review

Not commissioned, externally peer-reviewed.
